# Oxidative stress assessment by glutathione peroxidase activity and glutathione levels in response to selenium supplementation in patients with Mucopolysaccharidosis I, II and VI

**DOI:** 10.1590/1678-4685-GMB-2017-0334

**Published:** 2019-02-14

**Authors:** José Araújo de Oliveira-Silva, Joyce Umbelino Pinto Yamamoto, Renata Bernardes de Oliveira, Vaneisse Cristina Lima Monteiro, Beatriz Jurkiewcz Frangipani, Sandra Obikawa Kyosen, Ana Maria Martins, Vânia D’Almeida

**Affiliations:** 1 Universidade Federal de São Paulo Universidade Federal de São Paulo Centro de Referência em Erros Inatos do Metabolismo (CREIM) São PauloSP Brazil Centro de Referência em Erros Inatos do Metabolismo (CREIM), Universidade Federal de São Paulo, São Paulo, SP, Brazil; 2 Universidade Federal de São Paulo Universidade Federal de São Paulo Departmento de Psicobiologia Laboratório de Erros Inatos do Metabolismo (LEIM) São PauloSP Brazil Laboratório de Erros Inatos do Metabolismo (LEIM), Departmento de Psicobiologia, Universidade Federal de São Paulo, São Paulo, SP, Brazil

**Keywords:** Mucopolysaccharidosis, oxidative stress, selenium, glutathione peroxidase

## Abstract

We assessed levels of plasma selenium (Se), selenoproteins and their change after Se supplementation in patients with mucopolysaccharidosis (MPS) types I, II and VI. This was done in a retrospective study of the medical records of 30 patients with MPS I (n=13), MPS II (n=9) and MPS VI (n=8) who were being treated with enzyme replacement therapy. As part of routine nutritional monitoring, Se levels were measured, revealing that 28 patients (93.3%) had values below the normal range. Therefore, they received supplementation for 12 months, and Se was measured after 6 and 12 months. Glutathione peroxidase (GPx) activity, total glutathione (GSHt), oxidized glutathione (GSSG) and reduced glutathione (GSH) were measured at baseline and 6 months after Se supplementation. The mean GSHt at baseline was 7.90 ± 2.36 μmol/g Hb, and after Se supplementation it was 5.76 ± 1.13 μmol/g Hb; GSH/GSSG was 2.3 ± 1.16 at baseline and 0.58 ± 0.38 after supplementation. GPx activity was 16.46 ± 3.31 U/g Hb at baseline and 4.53 ± 4.92 U/g Hb after Se supplementation. The difference was shown to be statistically significant by paired *t*-test. In conclusion, our study demonstrated that oxidative stress parameters were altered by Se supplementation in patients with MPS I, II and VI who were previously deficient in Se.

## Introduction

Mucopolysaccharidoses (MPSs) are hereditary metabolic diseases caused by the deficiency in the activity of the lysosomal enzymes responsible for the degradation of glycosaminoglycans (GAGs). The storage of non-degraded or partially degraded GAGs compromises both the structure and function of cells and organs (Neufeld and Muenzer, 2001). MPSs are classified into 11 syndromes according to the deficient enzyme. The clinical manifestations are chronic and progressive, usually presenting a wide spectrum of severity depending on the enzyme deficiency (Neufeld and Muenzer, 2001; Wraith, 2006).

MPS I is inherited in an autosomal recessive trait, caused by mutations in the *IDUA* gene that encodes the enzyme alpha-L-iduronidase (EC 3.2.1.76), a lysosomal enzyme responsible for metabolizing the GAGs dermatan and heparan sulfate, and encompasses a spectrum of phenotypes that have been delineated into three separate diseases based on clinical presentation that are not distinguishable biochemically: the severe form, Hurler syndrome (OMIM: 607014), and the attenuated forms Hurler-Scheie (OMIM: 607015) and Scheie (OMIM 607016) (Neufeld, 2001).

MPS II, also known as Hunter syndrome (OMIM 309900), is an X-linked inherited trait, caused by mutations in the *IDS* gene that encode the enzyme iduronate 2-sulfatase (E.C. 3.1.6.13) leading to accumulation of dermatan and heparan sulfate in different organs and tissues (Neufeld and Muenzer, 2001). MPS VI, also known as Maroteaux-Lamy syndrome (OMIM 253200), is inherited in an autosomal recessive trait, caused by mutations in the *ARSB* gene that encodes the enzyme arylsulfatase B (E.C. 3.1.6.12), leading to accumulation of chondroitin sulfate in different organs and tissues (Neufeld and Muenzer, 2001).

Although biochemically distinct, MPS I, II and VI share some common clinical features such as hepatosplenomegaly, joint stiffness, dysostosis multiplex and cardiac alterations. Patients with the severe forms of MPS I and II present cognitive impairment and neurodegeneration as part of disease progression. Patients with MPS VI do not present cognitive impairment (Neufeld, and Muenzer, 2001).

It is estimated that the incidence for this group of diseases is 3.4 - 4.5 in 100,000 live births (Baehner *et al.*, 2005; Lin *et al.*, 2009). Though there is no specific treatment or cure for MPS, a range of possible treatments are being explored, including enzyme replacement therapy (ERT), which is currently only available for MPS I, MPS II, MPS IVA, MPS VI, and MPS VII (Saudubray *et al.*, 2006; Rohrbach and Clarke, 2007; [Bibr B17] Hendriksz *et al.*, 2015; FDA, 2017). Previous studies have shown that ERT helps to reduce the accumulation of GAGs in the organs, promoting a reduction in spleen and liver size, an improvement in growth rates, in walked distance measured by the 6-minute walking test, and in functional capacity (Decker *et al.*, 2010; Giugliani *et al.*, 2010).

Some studies report that there is an increase in oxidative stress in patients with MPSs, even in those receiving ERT, but the mechanisms of action remain largely unknown (Pereira *et al.*, 2008; Tessitore *et al.*, 2009; Jacques *et al.*, 2016). Oxidative stress is also common in neurodegenerative and non-neurodegenerative lysosomal storage diseases and is associated with a variety of diseases, including cancer and cardiovascular disease (Finkel and Holbrook, 2000; Dutta *et al.*, 2012).

There are a variety of defense systems against oxidative stress, including non-enzymatic antioxidants, such as melatonin, estrogens, bilirubin, reduced glutathione (GSH), polyphenols, and vitamins, in addition to antioxidant enzymes, such as superoxide dismutase (SOD), catalase (CAT), and glutathione peroxidase (GPx) (Tietze, 1969). The activity of GPx is dependent on selenium, which is an essential mineral in the diet due to the requirement for selenocysteine in some selenoproteins. GPx promotes protection against reactive oxygen species (ROS) and reactive nitrogen induced cell damage. Because of its antioxidant activity, there has been a great deal of interest in the study of Se and GPx (Halliwell and Gutteridge, 2007; Tinggi, 2008).

Our study aims to determine the levels of plasma selenium, oxidative stress status evaluated by the ratio of reduced glutathione to oxidized glutathione, GPx activity and the response to Se supplementation in patients with MPSs I, II and VI selected by convenience sampling.

## Materials and Methods

### Subjects

Patients with MPS I, II and VI who were receiving weekly ERT at the Reference Center for Inborn Errors of Metabolism (CREIM), Universidade Federal de São Paulo, São Paulo, Brazil were recruited for this study. All patients or their respective legal guardian read and signed an informed consent form. The study was approved by the Ethics Committee of the Universidade Federal de São Paulo under registration number 0763/11.

### Study design

A retrospective evaluation of medical records of patients with MPS I, II and VI. As part of the routine nutritional monitoring of patients with MPS, serum lipids, total protein, albumin, glucose, vitamin B12, vitamin D, folic acid, zinc and Se are assessed annually. In a cross-sectional retrospective analysis, it was noticed that the majority of patients had Se deficiency, and based on these results, we measured GPx activity, total glutathione (GSHt), oxidized glutathione (GSSG), and reduced glutathione (GSH) at baseline (T0). Se supplementation was then given for six months, and Se, GPx activity, GSHt and GSSG/GSH ratio were measured (T1). After another six months of Se supplementation (a total time of 12 months) Se levels were measured again (T2).

### Selenium supplementation

The selenium supplementation was based on the recommended dietary allowance (RDA) according to the age of the patients (20-55 μg/day), a daily value that would meet the mineral needs of 97-98% of the population (IOM 2000). The Se was supplied to the patients through a compounding pharmacy, formulated as syrup and was to be taken daily. Adherence to treatment was checked weekly/monthly.

The biochemical assays were carried out on freshly drawn blood samples and analyzed at the Laboratory of Inborn Errors of Metabolism (LEIM), Universidade Federal de São Paulo.

### Blood concentrations of Selenium, GSH/GSSG, and GPx activity

Plasma concentrations of Se were determined by hydride generation atomic absorption spectrometry (HG AAS) according to the method of Hao *et al.* (1996) using a Hitachi® Z-500 spectrometer. The results were expressed as μg/L.

Total glutathione and GSH concentrations were analyzed by high performance liquid chromatography (HPLC) through fluorescence detection and isocratic elution. The method used was that developed by Pfeiffer *et al.* (1999), with slight modifications: column C18 Luna (5 μm, 150 mm 4.6 mm), mobile phase (0.06 M sodium acetate, 0.5% acetic acid, pH 4.7 (adjusted with acetic acid, 2% methanol) and a flow rate of 1.1 mL/min. The retention time was 9 minutes for GSH.

For GSSG quantification, the method previously described for measuring erythrocyte GSH was used (Galdieri *et al.*, 2007), but without adding the reducing agent.

The determination of the erythrocyte GPx was performed in aliquots of the material obtained from the patients and spectrophotometrically analyzed using reduced nicotinamide adenine dinucleotide phosphate (NADPH) as a marker of the glutathione peroxidase activity. The reaction is based on the reduction of tert-butyl hydroperoxide by glutathione peroxidase, which uses NADPH to provide the reducing power in this reaction (Wahllander *et al.*, 1979). The results were expressed as μmol/g Hb for GSHt, GSSG, and GSH; for GPx they are given as U/g Hb.

### Statistical analysis

The quantitative data were evaluated for their internal consistency by the researchers before being included in the analysis. All data were presented as mean ± standard deviation (SD).

Differences in continuous variables, such as Se, GSH, GSSG, and GPx before and after supplementation were evaluated by Tukey’s multiple comparison test and, the dependencies between variables were calculated using the Pearson or Spearman coefficient of correlation. Also, the regression coefficient was calculated, and a 95% confidence interval (CI) for net misclassification was calculated using the bootstrap method with SPSS version 22.0 (IBM SPSS Statistics, New York, United States).

The significance of the differences between GPx, GSHt, and the GSH/GSSG ratio before and after Se supplementation was assessed by Student’s t-test and Paired *t*-test using Prism 5.0, GraphPad (San Diego, CA) software. A level of *p*<0.05 was accepted as statistically significant.

## Results

### Patient characteristics

Thirty patients were enrolled: 13 patients with MPS I (eight males and five females), nine patients with MPS II (eight males and one female), and eight with MPS VI (six males and 2 females). Mean age was 13.1 ± 8.3 years (range 3 – 30 y). The age of diagnosis, onset, and time of ERT are shown in Table 1.

**Table 1:** Demographic characterization of patients with MPS types I (n=13), II (n=9) and VI (n=8) enrolled in the study.

	MPS I (n=13)		MPS II (n=9)		MPS VI (n=8)	
	Mean ± SD	Median (min, max)	Mean ± SD	Median (min. max)	Mean ± SD	Median (min, max)
Age (y) at enrollment	18 ± 9.3	14.5 (6.6 - 30.3)	16.9 ± 8.7	13.2 (9.2 - 33.9)	16.4 ± 7.6	15.2 (7.2 - 28)
Age (y) at first medical consultation	10.2 ± 9.7	3.7 (0.5 - 24.1)	9.1 ± 9.1	4.9 (0.7 - 25.9)	4.4 ± 3.5	2.7 (1.5 - 10.5)
Age (y) at enzymatic diagnosis	10.7 ± 9.7	5.4 (0.4 - 27.1)	8.6 ± 9.2	4.1 (0.2 - 26)	6.1 ± 4.8	5.2 (1.6 - 14.2)
Age (y) at ERT initiation	11.1 ± 9.5	5.5 (1.3 - 25)	10.4 ± 8.7	6.3 (3.5 - 26.6)	10.1 ± 6.9	8.7 (2.7 - 20)
Time (y) under ERT	6.4 ± 1.9	5.7 (4.7 - 10)	6 ± 2.2	5.2 (3.4 - 11.2)	5.9 ± 1.5	5.6 (3.9 - 8.1)

SD: standard deviation; min: minimum and max: maximum.

### Selenium status before and after supplementation in MPSs patients

As part of the routine nutritional monitoring of MPS patients, Se levels were measured in 30 patients, showing that 28 patients (93.33%) were deficient with a mean of 35.7 ± 10.0 μg/L, and two were within the normal level (52 ± 1.13 μg/L) considering the laboratory reference value (46-143 μg/L). Thus, Se supplementation was given, adjusted to the required RDA according to age (T0).

**Figure 1:**
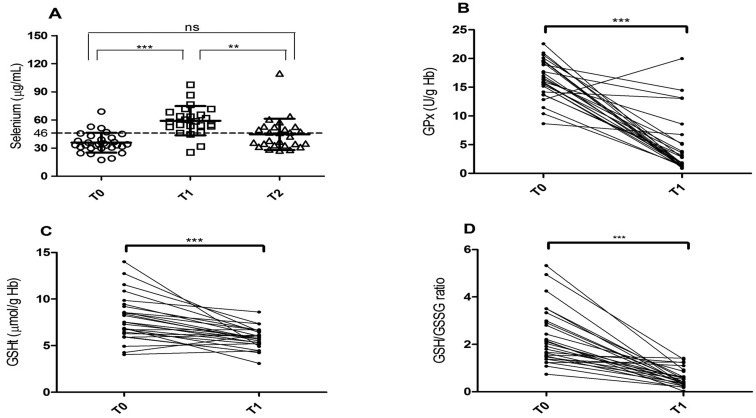
Selenium, GPx and GSHt activity. (A) Selenium levels in MPS patients at baseline (T0), 6 months (T1) and 12 months (T2) after supplementation. The dotted line means the cutoff value for normal Selenium level. Tukey’s Multiple Comparison Test: **p<0.01, ***p<0.001. (B) GPx activity at baseline (T0) and 6 months after (T1) selenium supplementation. (C) GSHt activity at baseline (T0) and 6 months after (T1) selenium supplementation. (D) GSH/GSSG ratio at baseline (T0) and 6 months after (T1) selenium supplementation. Paired *t*-test: **p<0.01, ***p<0.0001.

Selenium levels were measured after 6 (T1) and 12 months (T2). During this period, few patients were lost to follow-up due to transfer to another center, and the number of patients was therefore reduced in the later phases, meaning that only 24 patients underwent sample collection at T1 and 27 at T2. At T1, 21 out of 24 patients (87.5%) were in the normal Se level range (mean: 59.20 ± 15.6 μg/L) and 3 (12.5%) were below normal values (mean 33.67 ± 6.3 μg/L). At T2, the mean Se level was 44.63 ± 16.6 μg/L, with 13 out of 27 patients (54.1%) having values below normality, and the difference between T0 vs. T1, and T1 vs. T2 was statistically significant by Tukey’s multiple comparison test (Figure 1). Spearman’s correlation coefficient was not significant (*p* = 0.23) between T0 and T1, but was statistically significant between T1 and T2 (*p* = 0.035).

### Total glutathione, GSH/GSSG and GPx in MPSs patients

**Figure 2:**
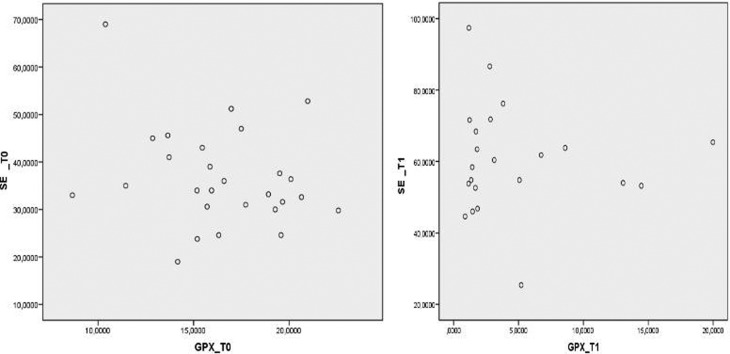
Scatter plot of GPx means on T0 and T1. Se_T0: selenium before supplementation; GPX_T0: glutathione peroxidase_ before supplementation. Se_T1: selenium after GPX_T1: glutathione peroxidase_ after supplementation.

Total glutathione (GSHt), reduced glutathione, and oxidized glutathione ratio (GSH/GSSG), as well as GPx activity were analyzed in only 27 patients, due to non-compliance to blood sample collection of all patients. Tests were performed twice, before (T0) and after six-months of Se supplementation (T1). GSHt mean concentrations before supplementation were 7.90 ± 2.36 and after they were 5.76 ± 1.13 μmol/g Hb. The mean GSH/GSSG ratio before supplementation was 2.34 ± 1.16 and after supplementation 0.58 ± 0.38. The mean value of GPx was 16.46 ± 3.31 U/g Hb before supplementation and after supplementation 4.53 ± 4.92 U/g Hb. The results by type of MPS are described in Table 2. The difference in GPx, GSHt, and GSH/GSSG ratio was shown to be statistically significant by paired Student’s *t*-test (*p*<0.0001) (Figure 1). No significant correlation was found between GPx activity and Se concentrations by Spearman’s test (*p* = 0.52), although there was a tendency to normality between GPx and patients supplemented with Se (Figure 2).

**Table 2:** GPx, GSHt and GSH/GSSG ratio of MPSs patients (n=27).

		MPS I	MPS II	MPS VI
		(n=13)	(n=7)	(n=7)
GSHt (μmol/g Hb)	*T0*	8.0 ± 1.8	8.11 ± 3.2	7.46 ± 2.7
	*T1*	5.85 ± 1.0	5.92 ± 1.3	5.44 ± 1.1
GSH/GSSG	*T0*	2.47 ± 1.3	2.36 ± 1.0	2.07 ± 1.0
	*T1*	0.7 ± 0.4	0.57 ± 0.3	0.38 ± 0.1
GPx (U/g Hb)	*T0*	15.8 ± 2.7	16.8 ± 4.5	17.36 ± 3.2
	*T1*	4.93 ± 6.2	3.5 ± 2.1	4.79 ± 4.9

## DISCUSSION

To the best of our knowledge this is the first study to address Se status, GSHt, GHS/GSSG, and GPx concentrations in response to Se supplementation in patients with MPSs I, II and VI. Despite the fact that MPS patients do not have to follow any specific or restricted diet, this data is important because of the high prevalence of Se deficiency (> 90%) that was observed. Selenium is an essential trace mineral that is of fundamental importance to human health. The first disease described caused by Se deficiency was Keshan disease, a potentially fatal form of cardiomyopathy, prevalent in children and endemic in parts of China (Rayman, 2000).

Some studies have shown that moderate selenium deficiency is linked to many conditions, such as increased cancer and infection risk, male infertility, decrease in immune and thyroid function, and several neurologic conditions, including Alzheimer’s and Parkinson’s disease (Papp *et al.*, 2007). Unlike in our study, in which all children and adolescents with MPS (n = 20) were Se deficient, a study (Vega *et al.*, 2017) using blood samples collected in northern Brazil did not find selenium deficiency in healthy children and teenagers. They attributed this fact to a diet rich in Brazil nuts and fish, foods commonly eaten in this region. In the present study, all the patients were from the Southeast region (São Paulo), which has different dietary habits and soil Se levels than the North. It should be noted that although the richest source of Se is Brazil nut, they are usually not part of the eating habits of the overall Brazilian population (Cozzolino, 2007).

A study of 66 preschool children enrolled in a public nursery school in São Paulo (Da Silva *et al.*, 2010) found that Se levels in the nails of the children aged from 2 to 6 years were in the normal range. These results suggest that preschool children living in the same region as the children in the present study did not have Se deficiency. A study with 81 adults (24 with thyroid dysfunctions and 57 from a control group) in the regions of São Paulo and Ceará also did not find Se and GPx deficiency (Maia CSC, Doctoral Thesis Universidade de São Paulo, São Paulo, Brazil). In our study, only two adult patients out of 10 were not Se deficient, however after Se supplementation there was an improvement in this condition.

Se supplementation can be used to treat the deficiency, as was shown in a study carried out in depleted patients in China (Yang *et al.*, 1989). A study in São Paulo showed that supplementation with Brazil nuts for at least four months increased Se status and GPx levels in capoeira practitioners (Coutinho., 2003). The concentration of GPx in plasma and erythrocyte, as well as enzymatic activity increased after supplementation with one Brazil nut daily (*p*<0.05), showing that this enzyme could be a marker of Se status (Coutinho, 2003). In the capoeira practitioners, enzyme activity was greater than in the control group, and the authors believe that the greater oxygen demand by the practitioners produces increased enzyme activity. This finding does not corroborate our results, in which GPx activity decreased after Se supplementation. We hypothesize that oxidative stress could be higher due to increased H_2_O_2_ production because of the depletion of Se (T0), requiring higher levels of enzyme activity. During the six months of supplementation there was a recovery in Se plasma levels, reducing H_2_O_2_ production and requiring less GPx enzyme activity, suggesting an adaptation of the enzyme to the biological system.

The relationship between plasma selenium levels and GPx activity is not always positively correlated, as was found in the present study. A high prevalence of Se deficiency (98.7%) was also found in a study about oxidative stress in hemodialysis patients (Pinto MBS, 2009, Doctoral Dissertation, Universidade de São Paulo, São Paulo, Brazil); however, only 11% of these patients presented reduced GPx values. After Se supplementation, GPx activity increased in all these patients (*p*<0.0001), unlike in our study. Mentro *et al.* (2005) evaluated the relationship between Se status, as measured by plasma, erythrocyte Se and GPx activity in 18 preterm infants (30 weeks gestational age) at risk of bronchopulmonary dysplasia. At postnatal weeks 1 and 4, selenium concentrations and GPx activity were measured and oxygen dependence and daily Se intakes were determined. Surprisingly, plasma and erythrocyte Se concentrations decreased from week one to week four despite routine nutritional Se intake, whereas erythrocyte GPx activity increased. The authors believe that the increase in erythrocyte GPx activity might be a response to oxidative stress and insufficient to counter the pulmonary oxidative damage induced by supplemental oxygen administration.

There are few studies of Se status in patients with MPS or other lysosomal diseases (LDs). Se deficiencies could result in a reduction in GPx and iodothyronine deiodinase (DIO) enzyme activities, and in increased production of H_2_O_2_, causing damage to the thyroid gland and impaired thyroid hormone metabolism (Contempre *et al.*, 1995). Most of the studies are about the Se-dependent enzyme glutathione peroxidase (GPx) and other enzymes involved in oxidative stress (SOD and CAT). These defense mechanisms to prevent or reduce the effects of oxidative stress depend on several dietary factors (Da Silva *et al.*, 2010). Lysosomes are highly susceptible to oxidative stress, and alterations that occur in this organelle due to the accumulation of GAGs in MPS I could increase their susceptibility to oxidative imbalance (Terman *et al.*, 2006). One of the possible hypotheses to explain the low levels of Se found in our study is that because it is a cofactor of GPx, which was higher, and considering that oxidative stress induces a cellular redox imbalance, over a long time this could have caused a depletion in the patients with MPSs, even those in ERT.

In a study that assessed the levels of the enzymes SOD, CAT and GSH in patients with MPS I (Pereira *et al.*, 2008), different enzyme levels were observed in patients receiving ERT compared to those who were not. CAT increased after four weeks of ERT, and SOD decreased after 12 weeks, but did not persist over the 24 following weeks. However, GSH levels did not decrease compared to the control group, indicating that GSH is not depleted in these patients. In the present study, we observed that the GSH and GPx levels decreased after Se supplementation, which could indicate a possible therapeutic effect in reducing the oxidative stress of these patients.

A study on MPS II (Filippon *et al.*, 2011) observed something similar before and after ERT in relation to SOD and CAT activities. Even during ERT, CAT activity showed a significant transient increase when compared to controls, returning to control values at the sixth month of ERT. There was no significant difference in SOD activity in pretreatment compared to controls, and no changes occurred during ERT, except for the sixth month, in which there was a significant increase in SOD activity, compared to controls. They concluded that ERT leads to a decrease in GAGs storage and that this may have restored some oxidative parameters. It is plausible to hypothesize that the accumulation of intralysosomal GAGs, directly or indirectly, may have an influence on the oxidative imbalance and possibly on the inflammatory process in MPS II patients. In the present study, 7 out of 8 patients with MPS II were Se deficient, and after supplementation the status of the mineral was recovered.

In another study (Jacques *et al.*, 2016), which included patients with MPS II, the authors did not find differences in SOD, CAT, GSH and GPx when compared with the control group. Moreover, they found that neither GSH content nor plasmatic antioxidant capacity (PAC) were reduced in patients, suggesting that there was no depletion of important non-enzymatic defenses. In our study, patients with MPS II showed a reduction in GPx, GSH and GSSG when supplemented with Se. We hypothesize that oxidative stress was higher (H_2_O_2_) when Se was depleted (T0), producing increased enzyme activity. After six months of Se supplementation, there was a recovery of plasma Se levels, H_2_O_2_ production was reduced, and less enzyme activity (GPx) was required, suggesting an adaptation of the enzyme to the biological system. Probably, this supplementation could help patients during ERT, since there is no consensus on how oxidative stress mechanisms interfere in the response to ERT.

There are few studies concerning oxidative stress in MPS patients. In one of them, several biomarkers were evaluated in 17 patients with MPS IV receiving ERT and compared with a healthy control group (n=10-15; Donida *et al.*, 2015). The concentration of erythrocyte GSH was significantly reduced in MPS IV patients when compared to the control group, indicating a reduced antioxidant defense, evidenced by a decrease in glutathione content and an increase in superoxide dismutase activity in erythrocytes. In the present study, there was a decrease in GSHt, GSH/GSS ratio, and GPx activity after Se supplementation in the MPS I, II and VI patients. Mean GSHt concentrations before supplementation were 7.90, and after they were 5.76 μmol/g Hb, while the GSH/GSSG ratio before supplementation was 2.3 and 0.58 after. In the Donida *et al.* (2015) study there were no significant differences in GPx activity between MPS IV patients (0.11 ± 0.006 U/mg protein) and controls (0.099 ± 0.006 U/mg protein). The mean value of GPx in the present study was 16.46 U/g Hb before supplementation and 4.53 U/g Hb after, and paired *t*-test showed the differences in GPx, GSHt and GSH/GSSG ratio to be statistically highly significant (*p*<0.0001). Despite the limitations of the present study, with no control group and only one enzyme for comparison, this data shows that even patients with MPS I, II and VI receiving ERT maintain some degree of oxidative stress. The study by Donida *et al.* (2015) reported that this was also the case with MPS IV patients.

In a study of MPS VI (Cé *et al.*, 2016), the degree of oxidative stress in MPS VI patients (n=8) was compared with MPS I patients (n=8) and a control group of healthy individuals (n=16) by SOD, CAT, and thiobarbituric acid reactive substances (TBARS) in plasma. The results showed that oxidative stress, evidenced by reduced CAT activity and greater TBARS production, was higher in the MPS groups compared to the control group.

Another study showed some altered biomarkers in MPS VI patients (Tessitore *et al.*, 2009). They found impaired autophagy, an accumulation of polyubiquitinated proteins, and mitochondrial dysfunction in fibroblasts from these patients. Similar alterations, along with inflammation and cell death, were observed in association with dermatan sulfate storage increase in the visceral organs of mucopolysaccharidosis VI mice (Tessitore *et al.*, 2009). In our study it was observed that all MPS VI patients were deficient in Se (n=8), even when compared with other types of MPS, and after supplementation they showed an improvement in GPx, GSH and GSSH status. Hence, despite the limitation in our study of not including a group control, this interventional and retrospective study showed for the first time a high prevalence of selenium deficiency in MPS patients and they could benefit from Se supplementation.

In conclusion, although oxidative stress in MPSs patients is not yet completely understood, our study demonstrated that oxidative stress parameters were altered by Se supplementation in patients with MPS I, II and VI who were previously deficient in Se.
